# Three-dimensional imaging of the forearm and hand: A comparison between two 3D imaging systems

**DOI:** 10.1371/journal.pdig.0000458

**Published:** 2024-04-18

**Authors:** Laura van Ginkel, Lotte Dupuis, Luc Verhamme, Erik Hermans, Thomas J. J. Maal, Vincent Stirler

**Affiliations:** 1 Department of Trauma Surgery, Radboud University Medical Center, Nijmegen, The Netherlands; 2 Technical Medicine, University of Twente, Enschede, The Netherlands; 3 Radboudumc 3D Lab, Radboud University Medical Center, Nijmegen, The Netherlands; National University Singapore Saw Swee Hock School of Public Health, SINGAPORE

## Abstract

The conventional treatment for distal radius fractures typically involves immobilization of the injured extremity using a conventional forearm cast. These casts do cause all sorts of discomfort during wear and impose life-style restrictions on the wearer. Personalized 3D printed splints, designed using three-dimensional (3D) imaging systems, might overcome these problems. To obtain a patient specific splint, commercially available 3D camera systems are utilized to capture patient extremities, generating 3D models for splint design. This study investigates the feasibility of utilizing a new camera system (SPENTYS) to capture 3D surface scans of the forearm for the design of 3D printed splints. In a prospective observational cohort study involving 17 healthy participants, we conducted repeated 3D imaging using both the new (SPENTYS) and a reference system (3dMD) to assess intersystem accuracy and repeatability. The intersystem accuracy of the SPENTYS system was determined by comparison of the 3D surface scans with the reference system (3dMD). Comparison of consecutive images acquired per device determined the repeatability. Feasibility was measured with system usability score questionnaires distributed among professionals. The mean absolute difference between the two systems was 0.44 mm (SD:0.25). The mean absolute difference of the repeatability of the reference -and the SPENTYS system was respectively 0.40 mm (SD: 0.30) and 0.53 mm (SD: 0.25). Both repeatability and intersystem differences were within the self-reported 1 mm. The workflow was considered easy and effective, emphasizing the potential of this approach within a workflow to obtain patient specific splint.

## Author summary

Within fracture care, conventional casts often induce patient discomfort and lifestyle restrictions, motivating our exploration of innovative alternatives. A promising solution involves the development of patient-specific 3D printed splints. These splints may offer enhancements such as improved aeration, reduced weight, and the ability to participate in water-related activities. The production of 3D splints typically starts with 3D imaging of the injured limb, which serves as input for subsequent splint design and the 3D printing process. Our research focuses on this initial phase by evaluating a new 3D imaging device.

Specifically, we investigate the feasibility of SPENTYS, a new portable 3D camera system tailored for surface scans of the limbs. Through a comparative analysis involving 17 healthy participants, we assess the SPENTYS accuracy and repeatability against a standard reference system within our hospital (3dmd). The results highlight SPENTYS’s accuracy, with a mean absolute difference of 0.44 mm compared to the reference system. Furthermore, positive feedback on usability from professionals underscores its potential in clinical practice.

## Introduction

Distal radius fracture (DRF) is a highly prevalent extremity injury in the Netherlands. [1]. The conventional treatment approach for these fractures includes immobilization of the injured extremity using a conventional forearm cast. While effective, these casts often lead to various discomforts and lifestyle restrictions on the wearer [2–5]. Examples are irritation and itching of the skin, pain from pressure points and inability to shower or swim without a protective sleeve.

The emergence of three-dimensional (3D) technology offers the potential for personalized splint design, which may address the drawbacks associated with conventional treatment. [6–12] To achieve this, commercially available 3D camera systems are utilized to capture patient extremities, generating 3D models for patient specific splint design. [10,13,14] design. The ability to use patient specific 3D printed splints in daily practice with a high quality fit relies, in part, on the capability to consistently and accurately capture of the limb using a 3D scanner. However, the intersystem accuracy of newly introduced 3D camera systems have not been investigated or compared to validated camera systems. Alongside considerations of system accuracy, it is essential to examine the repeatability and system usability, both of which are key determinants in the decision to integrate a 3D camera system into the patient-specific 3D printed splint workflow. This assessment represents a pivotal step towards the use of patient-specific 3D printed splints in daily practice.

The aim of this study is to investigate the accuracy and repeatability of a new mobile handheld camera system (SPENTYS system). This system is presumed to offer improved usability when compared to the more complex yet comprehensive and validated reference camera system, the 3dMD system, within the context of our hospital. The intersystem accuracy and repeatability between the SPENTYS system and the 3dMD system will be determined for a quantitative comparison between the two systems. The comparison study was limited to the SPENTYS and 3dMD system due to their relevance for inclusion in the 3D printed splint workflow and their availability in the hospital. We hypothesize that the SPENTYS system is accurate and repeatable (within a 1 mm range) in capturing 3D images of the forearm.

## Materials and methods

The study was approved by the local research ethics and clinical research committee (Research Ethics Committee of the Radboud University Nijmegen Medical Center, Nijmegen, the Netherlands; ID: 2020–6130, approval date: February 13^th^, 2020).

### Study design

We conducted a prospective observational cohort study. This report is written in compliance with the STROBE guidelines. In this study, 3D images of forearms and a phantom were acquired with the SPENTYS system and the already established 3dMD system. The primary study endpoints were the intersystem differences between images of both imaging systems and the repeatability of both imaging systems. The secondary outcome was the difference between the physical distances on a phantom and the digitally determined distances on the scans. This outcome was represented as and repeatability an absolute mean difference expressed in millimetres (mm). Another secondary outcome was the level of usability of the SPENTYS system measured as a System Usability Score (SUS).

### Setting

This study was conducted at the department of trauma surgery and the 3D Lab in the Radboud University Medical Center, Nijmegen, the Netherlands. This study was performed from June until August 2021. The 3D images were acquired by two commercially available imaging systems; the 3dMD system (3dMD cranial system, Atlanta, GA, USA) and the SPENTYS system (Spentys, Brussels, Belgium). The 3dMD system setup consisted of five pods with three cameras each. The SPENTYS system consisted of an Intel RealSense-D435 structure sensor camera (Intel Realsense, Santa Clara, California, USA) and an iPad camera (Apple, Cupertino, California, USA). The structure sensor uses structured light in order to capture depth data, using a frequency-matched infrared projector and sensor. The red-green-blue iPad camera captured surface color information.

**Fig 1:**
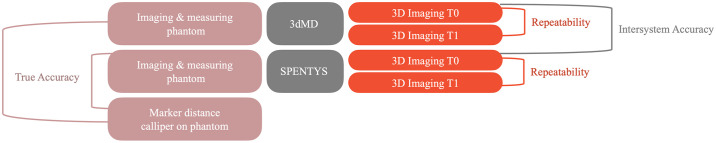
True accuracy, intersystem accuracy and repeatability measurements. Schematic overview of measurements performed using 3dMD and SPENTYS imaging systems.

**Fig 2:**
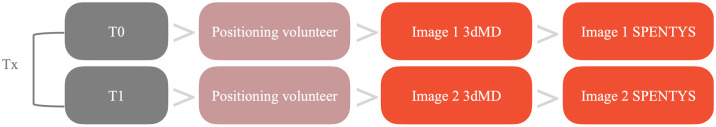
Repeatability and intersystem accuracy measurement sequence.

Measurements with the SPENTYS and 3dMD systems were performed in three different setups (Fig 1). A phantom was used in the first setup. It was generated using a 3dMD surface scan of a single individual. Three geometrical markers were added using Meshmixer (Autodesk Meshmixer, Inc. San Rafael, CA, USA). The phantom was printed with the Ultimaker PVA and Ultimaker PLA-Pearl white material using the Ultimaker S5 3D printer (Ultimaker B.V., Utrecht, Netherlands). The phantom (excluding the geometrical markers) was covered with a pantyhose to diminish scattering during image acquisition. Four 3D images of the prepositioned phantom were acquired by each imaging system. In the second setup, 3D images of both the left and right forearm and hand of participants were acquired with both camera systems at two separate points in time (T0 and T1) and with an interval of several minutes (Fig 2). All images were acquired by the same operator. The camera systems were calibrated according to the instructions provided by the manufacturers before image acquisition. Positioning of participants was identical during image acquisition with either system. The 3D images were taken with a participant seated in a 60 degrees flexion in the elbow joint using a static positioning tool. This level of flexion ensured the full functionality of all cameras of both systems. Moreover, the fingers were fixed in extension and attached to the metal 3dMD frame by cables. The participants were instructed to keep the arm and hand still.

In the last setup, evaluation of the usability of the SPENTYS-system was performed using a system usability scale (SUS). Nine participants (3 trauma surgeons, 3 plaster masters and 3 employees of the 3D Lab) were asked to perform multiple images of a propositioned volunteer using the SPENTYS system. The participants were asked to perform the image acquisition three times for training after a short briefing. Participants were then asked to perform the procedure without any support. The participants were asked to fill out a SUS questionnaire immediately after the last acquisition.

### Participants

A sample size was calculated based on an a priori power analysis (effect size 0.5, unreliability margin 0.05 and power of 0.95) to determine repeatability and intersystem accuracy of the 3D camera system of SPENTYS.

All participants were recruited at the department by asking to volunteer. Participants were included if they were eighteen years and older and without any pre-existent hand or arm deformity. Nine experts (3 trauma surgeons, 3 plaster masters and 3 employees of the 3D Lab) were selected to determine the usability.

### Measurements

**Fig 3:**
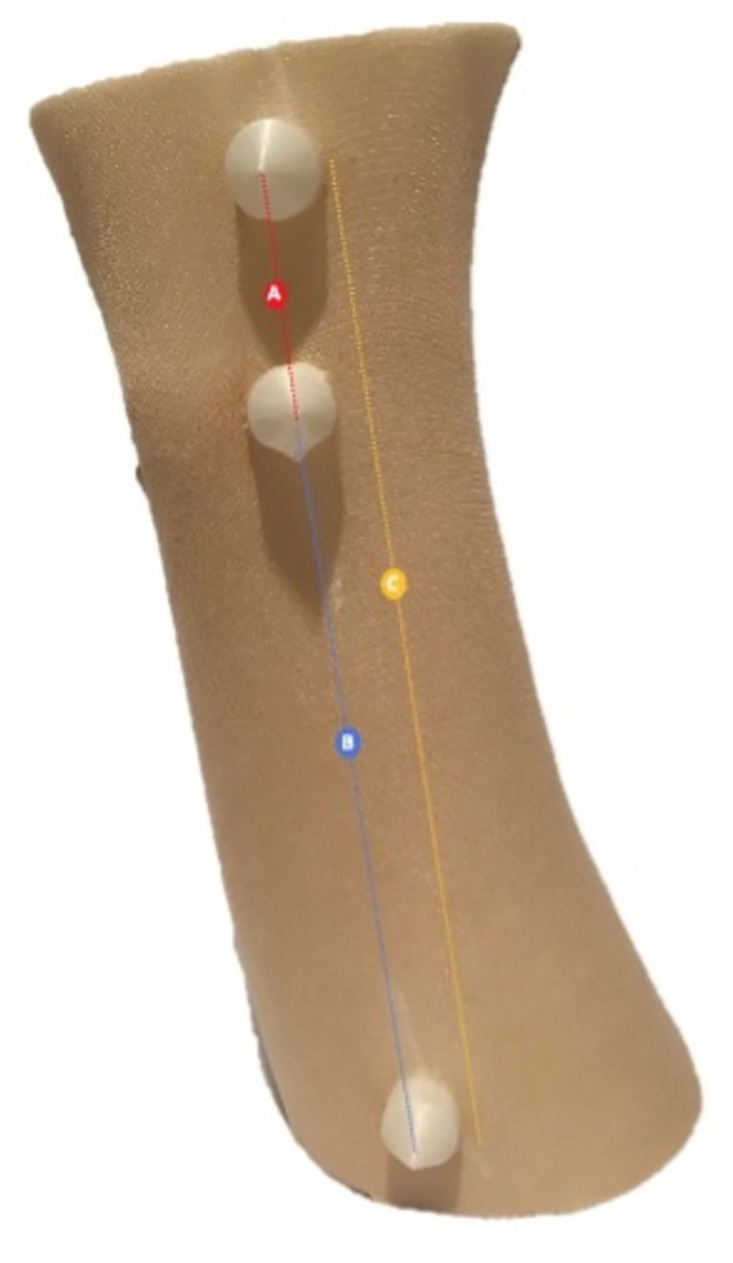
Three-dimensional printed phantom. Letters between geometrical markers indicate measurement paths.

Measurements calculated from the 3D images (3dMD and SPENTYS) were compared to calliper measurements for three measurements paths (Fig 3) on the phantom to obtain true accuracy. Physical path lengths were determined with a digital calliper (Mitutoya Digimatic, Aurora, USA). These measurements were repeated three times by the same observer to diminish intraobserver variation. Digital measurements were taken with the measure function of Solidworks (Version 2019, Dassault Systèmes SolidWorks Corporation, Waltman, MA, USA). All measurements were represented in mm.

**Fig 4:**
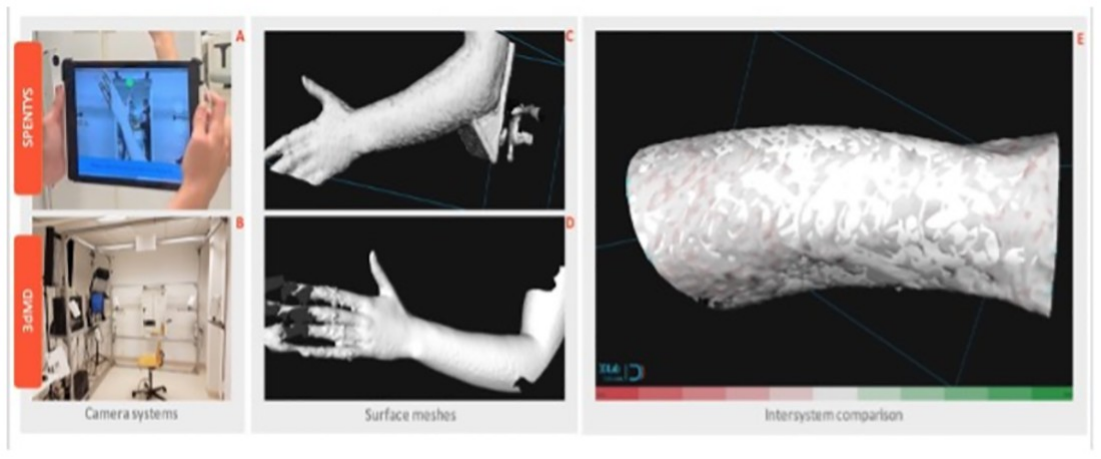
Intersystem accuracy. Two 3D imaging systems (A, B) with accompanying surface meshed (C, D). Section (E) shows the color-coded-heat map of the intersystem accuracy of the left arm of one of the particpants.

Intersystem accuracy was defined as the mean absolute difference between images, using the 3dMD system as a reference. Images taken at T0 were loaded into 3dMedX (version 1.2.17.0, 3D Lab Radboudumc, Nijmegen, Netherlands). An initial Procrustes analysis was performed to prealign both scans. Within 3dMedX both images were modified simultaneously. Regions proximal to the elbow joint were out of interest and thus removed. The fingers and hands were removed to reduce potential incongruencies due to movement. The modified SPENTYS image was matched to the modified 3dMD image using the iterative closest point algorithm (ICP). Subsequently, the distance from each point on the first image towards the closest point of the second image was calculated. Differences (represented in mm) were visualised with a color-coded heat map (Fig 4). The first image (T0) of each system was matched to the second image (T1) to determine repeatability in a similar way to the intersystem accuracy. Differences were also visualised with a color-coded heat map. Analyses that could not be performed due to missing data were omitted. The usability of the SPENTYS system was assessed with a 10-item questionnaire on a 5-point Likert scale. A SUS score above 68 was considered above average [15].

### Statistical analysis

Statistical analyses were performed in MATLAB (R2021a, MathWorks, California, USA) and the statistical toolbox of 3dMedX. Assumptions of normality were assessed by visual inspection of histograms. All outcomes were measured on a continuous scale, except for the system usability score.

## Results

### True accuracy

**Fig 5:**
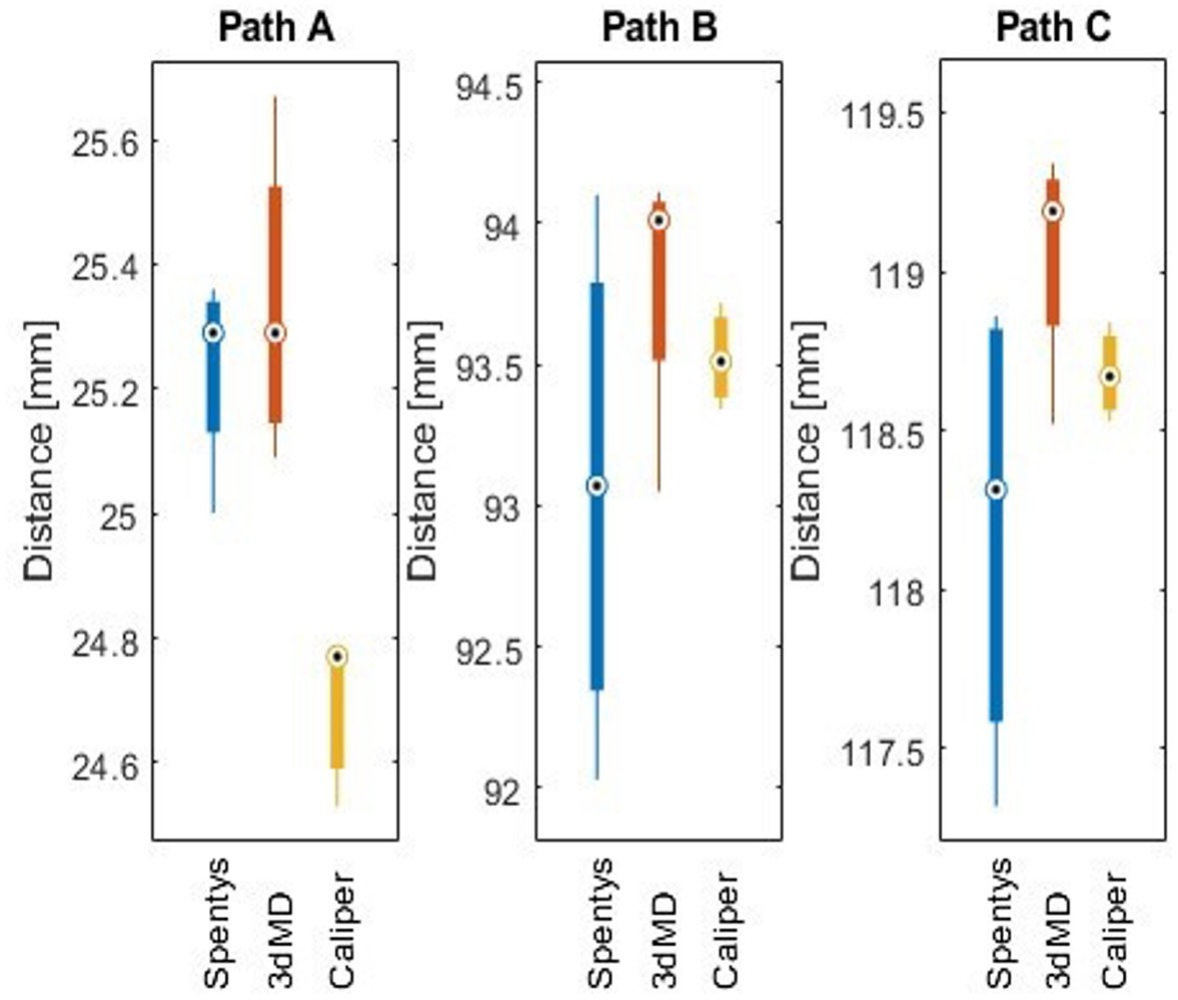
Boxplots of mean distances. Mean distances measured by the SPENTYS system, 3dMD 186 system and digital calliper. The mean and variance are given for measurement paths A, B and 187 C of the phantom.

The several calibrations of both systems all passed the calibration test and no calibration errors were reported during measurements. The true accuracy for measurement paths A, B and C are represented in Fig 5. The absolute mean differences of the SPENTYS and the 3dMD systems showed approximately equal values. The SPENTYS system showed a larger variety of distances at measurement paths B and C. Moreover, the SPENTYS system showed a smaller variation of distance at measurement path A. The measurements showed similar results for both imaging systems and results were comparable to the calliper measurements.

### Intersystem accuracy and repeatability

**Fig 6:**
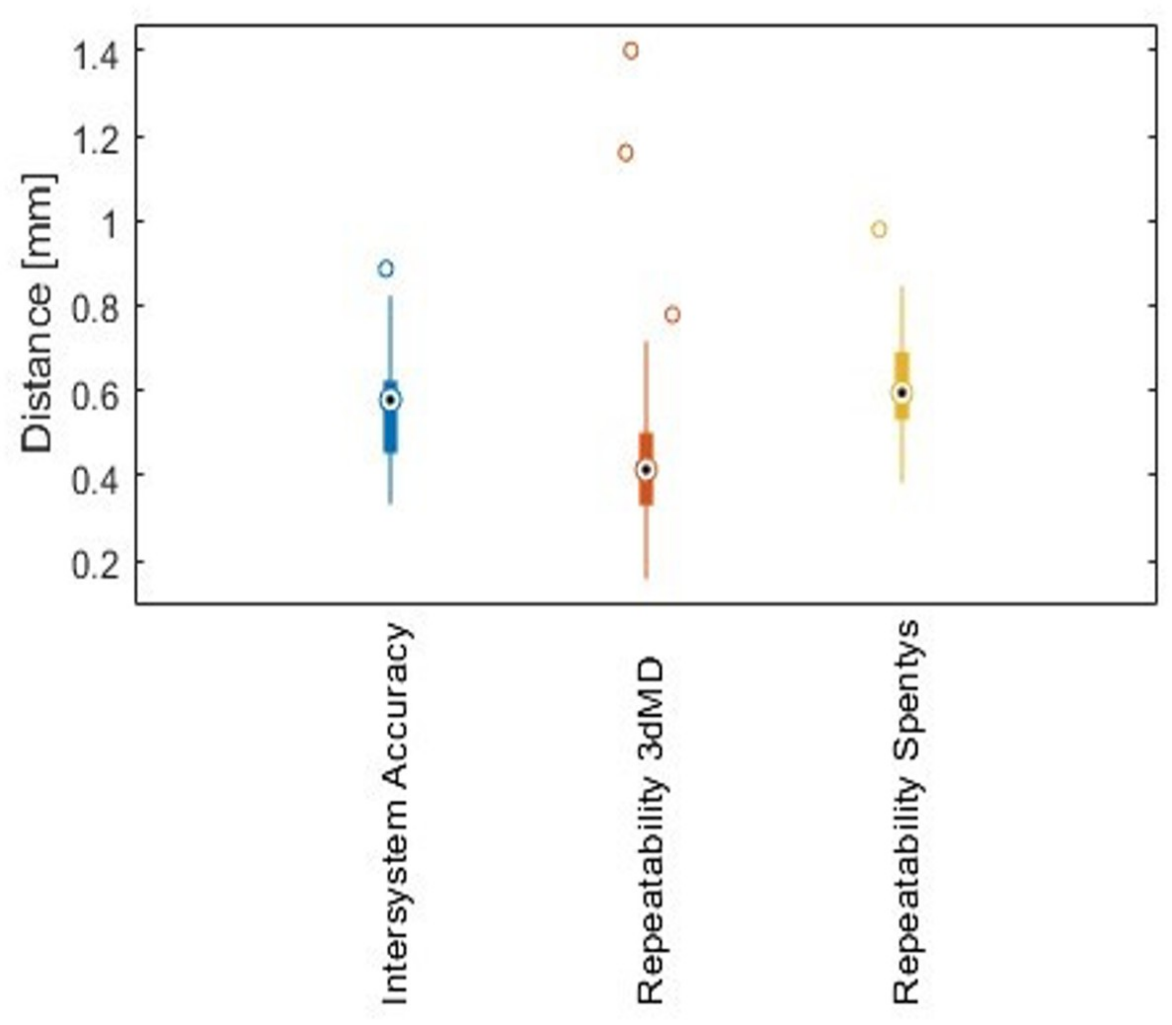
Boxplots of the intersystem accuracy and repeatability. Outcomes of both 3D imaging 207 systems are represented in mm. Outliers are represented by circles.

Seventeen healthy participants (nine males and eight females) with a mean age of 23.7 y 191 (range 22- 46y) were included. The mean absolute difference between the SPENTYS and 3dMD 192 systems was 0.44 ± 0.25 mm (p = 0,0001) (Fig 6). The mean absolute difference of the repeatability 193 of the SPENTYS system and 3dMD system was respectively and 0.53 ± 0.25 mm (p = 0,0001) 194 and 0.41 ± 0.30 mm (p = 0,0001) (Fig 6). Both repeatability and intersystem differences were within 195 the 1 mm range and the differences were considered negligible from a clinical point of view.

### Usability

A SUS of 68 is considered ‘above average’ (15).The mean SUS of this study was 76 (range 40–95).

## Discussion

### True accuracy

To the best of our knowledge, no 3D camera system has been described within literature as a gold standard for 3D image acquisition of the forearm and hand. The 3dMD system was considered the reference system in this research due to its clinical application within our hospital and coverage in the literature for other anatomical regions [14,16,17].Measurements on a phantom were performed to determine the true accuracy. Results showed that neither one of the 3D imaging systems was superior to the other. There are some limitations in the determination of the true accuracy. The geometrical markers were added collinear to each other and perpendicular to the phantom surface. Ideally, the markers are more randomly distributed on the phantom. However, to ease the physical measurements collinear markers were chosen. Prior versions of the phantom showed that the smooth phantom surface scattered light, introducing stitching errors and noise. Therefore, the surface of the phantom was covered with a pantyhose.

### Intersystem accuracy and repeatability

The intersystem accuracy was investigated through the difference between matched images captured by the SPENTYS and 3dMD systems. The SPENTYS system produced significantly different images compared to the 3dMD system with a statistical null hypothesis equal to zero. However, differences were negligible from a clinical point of view because the determined intersystem accuracy was within the reported 1 mm limit [12]. One outlier for the determination of the intersystem accuracy was seen. This may have emerged due to an arm position change in the time between the captions of the 3dMD system and the SPENTYS system. The absolute mean difference of 0.89 mm of this outlier was still within the limit of 1 mm. The repeatability was measured by comparison of consecutive images acquired per imaging system. The 3dMD system had a median of 0.41 mm and showed a slightly better repeatability than the SPENTYS system, with a median of 0.59 mm. The repeatability outcomes of the 3dMD and SPENTYS system showed respectively three and one outliers. Two of the 3dMD outliers were outside the 1 mm range. Both the 3dMD- and SPENTYS system showed an outlier for imaging the left arm of participant 7. This was probably caused by positioning changes during data acquisition. After inspection of the surface meshes no indication was found for the two other outliers of the 3dMD system. Given the repeatability of both systems, not one of the systems was considered superior to the other. Daemen et al (2021) conducted a comparison between three different imaging systems for imaging the chest, including the 3dMD system, the Artec Leo and the EinScan Pro 2X Plus. They reported a mean absolute difference for the repeatability of the 3dMD system of 0.59 mm (SD = 1.05mm) and a true accuracy (versus calliper) of 0.89 mm. Both values are in the same order of magnitude as the repeatability found within this study [12].

To determine repeatability and intersystem accuracy an image set was matched. This gives the advantage to apply two plane cuts at the same location in both images. Reduction of motion artefacts is essential for a precise determination of true accuracy or repeatability. Therefore, images of an image set need to be captured in the most identical position during data acquisition. Within this research, Chinese fingers and a self-made positioning tool were used to ensure a participant’s position. However, this cannot prevent all motion. We chose to reduce the influence of motion by removing the hand and fingers of the surface mesh during data analyses. 3D printed splints are aimed at covering the forearm and a part of the hand. A potential limitation of this study is, therefore, that we did not consider the complete region of interest for personalized splint design. This limitation will not influence the characteristics of the 3D camera system itself. However, we cannot omit that obtained 3D models are affected when the region of the hands is included in post-processing. The fact that only healthy adult participants were included may be regarded as a limitation. Although children are a potential group for treatment with 3D printed splints, it was deliberately chosen only to enrol adult participants because no difference in accuracy and repeatability is expected from a technical point of view. Automatic recognition of the region of interest for ICP matching can be considered as an improvement. Future research seeking to use this methodology should attempt to find a way to reduce hand movement even more.

### Usability

A secondary endpoint was the usability of the SPENTYS system. The mean SUS in this study was 76 (range 40–95). The usability of this system was, to our knowledge, not covered in the literature. Therefore, we were unable to directly compare our results with other series. None of the trauma surgeons, plaster masters and employees of the 3D Lab reported relatively low or high SUS. However, only a small population participated. No accompanying software for personalized 3D splint design is available for the 3dMD system. The SUS was, therefore, not determined for the 3dMD system.

### Clinical relevance

It is essential to point out that the comparative study of two 3D camera systems is not engaging in itself. However, it represents the first step for a potential implementation of personalized 3D splints within the clinical workflow. The 3D SPENTYS camera system showed to be accurate in 3D imaging of the forearm and hand in comparison with a clinical validated 3dMD system. The reported statistically significant differences in measured differences are not relevant from a clinical point of view, because the differences are below 1 mm.

## Conclusion

3D imaging of the forearm seems feasible as a cornerstone for patient specific 3D printed splint design. The SPENTYS system showed comparable repeatability to the 3dMD reference system. The intersystem accuracy high and well below the reported limit of 1 mm. According to the SUS scale, the SPENTYS system seems to have a good practical use among trauma surgeons, plaster masters and employees of the 3D Lab. Consequently, the Spentys system erges as a suitable option to meet the prerequisites of a 3D workflow tailored for the production of patient-specific 3D printed splints.

## Supporting information

S1 Data Table 1: Intersystem accuracy per patient and scanned arm. Table 2: Repeatability 3dMD system per patient and scanned arm. Table 3: Repeatibility 3dMD system per patient and scanned arm. Figures: Histograms of the Mean absolute difference of Intersystem accuracy and 3D scanner Repeatability.
(PDF)
