# Supplementary material for: Three-dimensional imaging of the forearm and hand: A comparison between two 3D imaging systems
Source: PLOS Digit Health. 2024 Apr 18;3(4):e0000458. doi: 10.1371/journal.pdig.0000458 (PMC11025912; doi:10.1371/journal.pdig.0000458)
Supplement: S1 Data — Table 1: Intersystem accuracy per patient and scanned arm. Table 2: Repeatability 3dMD system per patient and scanned arm. Table 3: Repeatibility 3dMD system per patient and scanned arm. Figures: Histograms of the Mean absolute difference of Intersystem accuracy and 3D scanner Repeatability. (PDF) [file pdig.0000458.s001.pdf]

Appendix B

	Minimal Value	5th Percentile	10th Percentile	median (50th percentile)	90th Percentile	95th Percentile	Maximal Value	Mean Value	Standard Deviation	RMS	Absolute Mean Value	Absolute Standard Deviation	Set Minimum Range
**P1.L1**	-3.96	-1.12	-0.72	0.14	1.04	1.35	4.12	0.14	0.8	0.81	0.59	0.8	-5
**P1.R1**	-3.99	-1.63	-1.19	-0.09	0.9	1.26	4.99	-0.09	1.02	1.02	0.72	1.02	-5
**P2.L1**	-5	-1.43	-0.85	0.22	1.51	2.11	4.98	0.27	1.13	1.16	0.82	1.13	-5
**P2.R1**	-2.59	-1.06	-0.85	-0.21	0.34	0.49	1.41	-0.24	0.48	0.54	0.41	0.48	-5
**P3.L1**	-3.06	-1.16	-0.91	-0.23	0.36	0.53	1.93	-0.26	0.54	0.6	0.45	0.54	-5
**P3.R1**	-1.95	-0.77	-0.61	-0.05	0.5	0.65	1.35	-0.06	0.43	0.44	0.35	0.43	-5
**P4.L1**	-4.2	-1.22	-0.86	0.22	2.07	2.87	5	0.39	1.2	1.26	0.89	1.2	-5
**P4.R1**	-1.75	-0.75	-0.6	-0.08	0.44	0.57	1.64	-0.08	0.41	0.42	0.33	0.41	-5
**P5.L1**	-4.09	-1.04	-0.66	0.2	1.03	1.39	4.96	0.22	0.88	0.9	0.62	0.88	-5
**P5.R1**	-2.41	-1.16	-0.91	-0.15	0.64	0.92	2.82	-0.13	0.65	0.66	0.51	0.65	-5
**P6.L1**	-2.14	-0.72	-0.52	0.2	1.22	1.87	4.66	0.32	0.83	0.89	0.6	0.83	-5
**P6.R1**	-1.84	-0.92	-0.74	-0.13	0.44	0.59	1.38	-0.14	0.46	0.48	0.39	0.46	-5
**P7.L1**	-4.05	-1.36	-0.79	0.22	1.36	2.2	4.9	0.27	1.12	1.15	0.77	1.12	-5
**P7.R1**	-2.28	-0.98	-0.77	-0.14	0.46	0.62	2.32	-0.15	0.49	0.51	0.41	0.49	-5
**P8.L1**	-3.2	-0.9	-0.67	0.15	1.14	1.74	4.82	0.25	0.87	0.91	0.62	0.87	-5
**P8.R1**	-1.65	-0.77	-0.62	-0.05	0.51	0.66	1.42	-0.05	0.44	0.44	0.35	0.44	-5
**P9.L1**	-1.99	-0.82	-0.6	0.21	1.16	1.48	4.55	0.25	0.71	0.76	0.58	0.71	-5
**P9.R1**	-4.4	-1.81	-1.38	-0.23	0.52	0.74	1.89	-0.34	0.76	0.83	0.61	0.76	-5
**P10.L1**	-4.83	-0.81	-0.57	0.23	1.14	1.55	4.67	0.27	0.74	0.79	0.59	0.74	-5
**P10.R1**	-2.71	-1.06	-0.82	-0.02	0.76	1	2.43	-0.03	0.63	0.63	0.5	0.63	-5
**P11.L1**	-2.51	-0.85	-0.61	0.19	1.24	1.78	4.78	0.29	0.84	0.89	0.63	0.84	-5
**P11.R1**	-4.25	-2.03	-1.39	-0.19	0.5	0.67	2.48	-0.33	0.79	0.86	0.6	0.79	-5
**P12.L1**	-3.91	-1	-0.75	0.1	1.1	1.64	4.92	0.2	0.91	0.93	0.64	0.91	-5
**P12.R1**	-2.47	-1.42	-1.18	-0.24	0.62	0.82	4.83	-0.27	0.69	0.74	0.59	0.69	-5
**P13.L1**	-2.59	-0.88	-0.6	0.17	1.19	1.63	3.06	0.23	0.75	0.78	0.58	0.75	-5
**P13.R1**	-3.98	-2.07	-1.54	-0.25	0.45	0.63	1.57	-0.4	0.8	0.89	0.63	0.8	-5
**P14.L1**	-2.5	-0.72	-0.51	0.21	1.09	1.43	4.84	0.28	0.72	0.77	0.55	0.72	-5
**P14.R1**	-2.1	-0.99	-0.78	-0.13	0.46	0.6	1.35	-0.15	0.48	0.51	0.4	0.48	-5
**P15.L1**	-1.75	-0.61	-0.45	0.13	0.81	1.09	2.35	0.16	0.52	0.54	0.41	0.52	-5
**P15.R1**	-4.55	-1.78	-1.26	-0.2	0.51	0.72	3.85	-0.3	0.76	0.82	0.59	0.76	-5
**P16.L1**	-2.36	-0.89	-0.66	0.12	0.97	1.25	3.08	0.14	0.66	0.68	0.52	0.66	-5
**P16.R1**	-2.62	-1.34	-1.09	-0.16	0.59	0.82	2.31	-0.2	0.66	0.69	0.54	0.66	-5
**P17.L1**	-4.99	-0.66	-0.45	0.23	1	1.33	3.74	0.25	0.65	0.7	0.51	0.65	-5
**P17.R1**	-4.92	-1.84	-0.99	-0.04	0.57	0.71	1.44	-0.17	0.76	0.78	0.51	0.76	-5

Table 1: Intersystem accuracy

Appendix C

	Minimal Value	5th Percentile	10th Percentile	median (50th percentile)	90th Percentile	95th Percentile	Maximal Value	Mean Value	Standard Deviation	RMS	Absolute Mean Value	Absolute Standard Deviation	Set Minimum Range
**P1.L**	-4.07	-2.25	-1.96	-0.58	1.61	2.31	5	-0.37	1.38	1.43	1.16	1.38	-5
**P1.R**	-1.61	-0.56	-0.43	-0.01	0.45	0.62	1.36	0	0.34	0.34	0.26	0.34	-5
**P2.L**	-1.87	-0.81	-0.63	0.07	0.85	1.04	2	0.09	0.57	0.58	0.48	0.57	-5
**P2.R**	-1.96	-1.13	-0.88	-0.05	0.59	0.77	3.1	-0.11	0.58	0.59	0.45	0.58	-5
**P3.L**	-1.96	-1.13	-0.88	-0.05	0.59	0.77	3.1	-0.11	0.58	0.59	0.45	0.58	-5
**P3.R**	-2.43	-0.99	-0.65	-0.02	0.38	0.49	1.53	-0.09	0.45	0.46	0.33	0.45	-5
**P4.L**	-2.79	-1.05	-0.81	-0.07	0.35	0.45	1.06	-0.16	0.48	0.51	0.35	0.48	-5
**P4.R**	-2.4	-0.82	-0.58	0.03	0.47	0.63	1.32	-0.01	0.43	0.43	0.34	0.43	-5
**P5.L**	-2.04	-0.8	-0.62	0.01	0.62	0.97	1.88	-0.01	0.52	0.52	0.37	0.52	-5
**P5.R**	-1.34	-0.52	-0.41	0	0.49	0.93	1.86	0.04	0.44	0.44	0.31	0.44	-5
**P6.L**	-1.47	-0.82	-0.67	-0.01	1.14	1.5	2.24	0.1	0.68	0.69	0.52	0.68	-5
**P6.R**	-2.12	-0.75	-0.55	0.01	0.56	0.83	2.13	0.01	0.51	0.51	0.37	0.51	-5
**P7.L**	-1.94	-0.93	-0.74	0.05	2.15	2.77	4.1	0.33	1.1	1.15	0.78	1.1	-5
**P7.R**	-3.71	-1.09	-0.8	-0.09	0.65	0.85	2.32	-0.09	0.63	0.64	0.48	0.63	-5
**P8.L**	-2.34	-0.64	-0.53	-0.04	0.54	0.7	1.99	-0.01	0.43	0.43	0.34	0.43	-5
**P8.R**	-1.03	-0.35	-0.25	-0.01	0.23	0.29	0.94	-0.01	0.2	0.2	0.16	0.2	-5
**P9.L**	-4.99	-1.87	-1.07	-0.02	1.06	1.48	3.97	-0.04	1.03	1.03	0.72	1.03	-5
**P9.R**	-1.31	-0.78	-0.67	-0.02	1.27	1.97	4.63	0.18	0.84	0.85	0.62	0.84	-5
**P10.L**	-4.14	-0.85	-0.68	0	0.62	0.74	1.52	-0.02	0.5	0.5	0.4	0.5	-5
**P10.R**	-1.88	-0.6	-0.38	-0.02	0.31	0.42	1.1	-0.04	0.32	0.33	0.23	0.32	-5
**P11.L**	-1.73	-0.91	-0.71	0	0.69	0.85	1.63	-0.02	0.55	0.55	0.44	0.55	-5
**P11.R**	-2.02	-1.14	-0.95	0.02	1	1.38	2.97	0.03	0.77	0.77	0.62	0.77	-5
**P12.L**	-3.17	-1.21	-0.86	0.01	1.03	1.42	4.35	0.04	0.86	0.86	0.63	0.86	-5
**P12.R**	-3.08	-0.71	-0.54	-0.04	0.56	0.76	1.9	-0.03	0.43	0.43	0.32	0.43	-5
**P13.L**	-1.08	-0.59	-0.44	0.09	0.64	0.87	2.14	0.1	0.44	0.45	0.34	0.44	-5
**P13.R**	-3.34	-1.18	-0.85	0.11	0.6	0.76	1.78	-0.02	0.62	0.62	0.44	0.62	-5
**P14.L**	-1.22	-0.63	-0.45	-0.03	0.41	0.57	1.42	-0.03	0.35	0.35	0.27	0.35	-5
**P14.R**	-0.84	-0.33	-0.28	-0.02	0.24	0.31	0.82	-0.02	0.2	0.2	0.16	0.2	-5
**P15.L**	-1.29	-0.61	-0.52	-0.04	0.77	1.05	1.71	0.05	0.51	0.51	0.41	0.51	-5
**P15.R**	-1.59	-0.36	-0.28	-0.01	0.3	0.44	1.01	0	0.25	0.25	0.19	0.25	-5
**P16.L**	-2.03	-0.96	-0.68	-0.03	0.65	0.88	2.43	-0.02	0.57	0.57	0.42	0.57	-5
**P16.R**	-5	-3.45	-2.66	-0.44	1.38	1.82	5	-0.56	1.65	1.74	1.4	1.65	-5
**P17.L**	-2.58	-1.27	-0.9	-0.03	0.67	0.85	1.7	-0.08	0.64	0.64	0.5	0.64	-5
**P17.R**	-1.73	-0.8	-0.67	-0.07	0.67	0.82	1.6	-0.03	0.5	0.51	0.42	0.5	-5

Table 2: repeatability 3dMD system

Appendix D

	Minimal Value	5th Percentile	10th Percentile	median (50th percentile)	90th Percentile	95th Percentile	Maximal Value	Mean Value	Standard Deviation	RMS	Absolute Mean Value	Absolute Standard Deviation	Set Minimum Range
**P1.L**	-4.59	-1.53	-1.2	-0.03	1.05	1.35	3.28	-0.06	0.89	0.89	0.7	0.89	-5
**P1.R**	-3.89	-1.4	-1.08	-0.16	0.73	0.96	2.56	-0.18	0.73	0.75	0.59	0.73	-5
**P2.L**	-2.73	-1.41	-1.05	0.06	1.15	1.54	3.88	0.06	0.88	0.89	0.69	0.88	-5
**P2.R**	-3.16	-1.41	-1.09	0.07	1.08	1.42	4.1	0.05	0.86	0.86	0.67	0.86	-5
**P3.L**	-3.34	-1.25	-1	-0.07	0.75	0.98	4.95	-0.09	0.72	0.72	0.55	0.72	-5
**P3.R**	-3.28	-1.32	-0.97	-0.07	0.79	1.06	4.48	-0.07	0.78	0.78	0.58	0.78	-5
**P4.L**	-4.92	-2.23	-1.49	-0.04	1.01	1.51	3.55	-0.15	1.1	1.11	0.8	1.1	-5
**P4.R**	-2.11	-0.99	-0.75	0.05	0.76	0.97	2.37	0.03	0.61	0.61	0.48	0.61	-5
**P5.L**	-2.9	-1.15	-0.94	-0.14	0.85	1.31	4.66	-0.06	0.82	0.82	0.6	0.82	-5
**P5.R**	-1.91	-0.86	-0.66	0.09	1.09	1.37	3.37	0.16	0.69	0.7	0.55	0.69	-5
**P6.L**	-3.31	-1.25	-0.98	0.01	1.03	1.37	4.93	0.02	0.8	0.8	0.64	0.8	-5
**P6.R**	-3.19	-1.16	-0.85	0	0.8	1.03	2.41	-0.03	0.69	0.69	0.53	0.69	-5
**P7.L**	-4.87	-1.9	-1.6	-0.15	1.47	1.93	3.7	-0.13	1.21	1.22	0.98	1.21	-5
**P7.R**	-4.78	-1.39	-0.98	-0.02	1.02	1.3	3.76	-0.04	0.91	0.91	0.66	0.91	-5
**P8.L**	-3.07	-1.33	-1.05	-0.09	0.76	1.03	4.47	-0.12	0.75	0.76	0.58	0.75	-5
**P8.R**	-1.88	-0.93	-0.75	-0.05	0.71	0.99	2.82	-0.02	0.6	0.6	0.47	0.6	-5
**P9.L**	-4.59	-1.9	-1.45	-0.11	1.12	1.51	3.7	-0.14	1.05	1.06	0.81	1.05	-5
**P9.R**	-2.13	-1.04	-0.82	0.11	1.45	1.87	4.85	0.22	0.89	0.92	0.71	0.89	-5
**P10.L**	-3.11	-1.21	-0.95	0.02	1.1	1.37	2.5	0.05	0.79	0.79	0.64	0.79	-5
**P10.R**	-3.05	-1.22	-1.01	-0.21	0.58	0.79	1.61	-0.22	0.64	0.67	0.53	0.64	-5
**P11.L**	-2.61	-1.4	-1.17	-0.06	1.06	1.38	2.85	-0.06	0.86	0.86	0.7	0.86	-5
**P11.R**	-3.5	-1.82	-1.49	-0.08	1.19	1.48	2.81	-0.13	1.02	1.03	0.85	1.02	-5
**P12.L**	-2.21	-0.97	-0.76	0.02	0.76	0.97	2.9	0.01	0.6	0.6	0.47	0.6	-5
**P12.R**	-2.21	-0.97	-0.76	0.02	0.76	0.97	2.9	0.01	0.6	0.6	0.47	0.6	-5
**P13.L**	-2.13	-1.02	-0.8	0.08	0.9	1.11	2.29	0.06	0.65	0.65	0.53	0.65	-5
**P13.R**	-2.57	-1.3	-0.94	0.16	1.08	1.31	2.71	0.11	0.79	0.8	0.65	0.79	-5
**P14.L**	-2	-1.08	-0.88	-0.11	0.82	1.12	2.82	-0.05	0.68	0.68	0.54	0.68	-5
**P14.R**	-2.24	-0.8	-0.6	0.02	0.62	0.81	1.74	0.02	0.48	0.48	0.38	0.48	-5
**P15.L**	-2.32	-1.03	-0.81	0	0.9	1.15	2.77	0.02	0.66	0.66	0.53	0.66	-5
**P15.R**	-2.41	-1.01	-0.8	0.02	0.94	1.22	4.17	0.05	0.69	0.69	0.54	0.69	-5
**P16.L**	-2.55	-1.07	-0.86	0.03	0.93	1.18	3.25	0.04	0.69	0.69	0.56	0.69	-5
**P16.R**	-3.17	-1.69	-1.4	-0.23	0.87	1.49	4.35	-0.19	1	1.02	0.76	1	-5
**P17.L**	-3.02	-1.35	-1.03	-0.04	0.99	1.25	2.15	-0.04	0.78	0.79	0.63	0.78	-5
**P17.R**	-3.12	-1.38	-1.08	-0.01	0.91	1.15	2.46	-0.05	0.77	0.77	0.62	0.77	-5

Table 3: repeatability 3dMD system

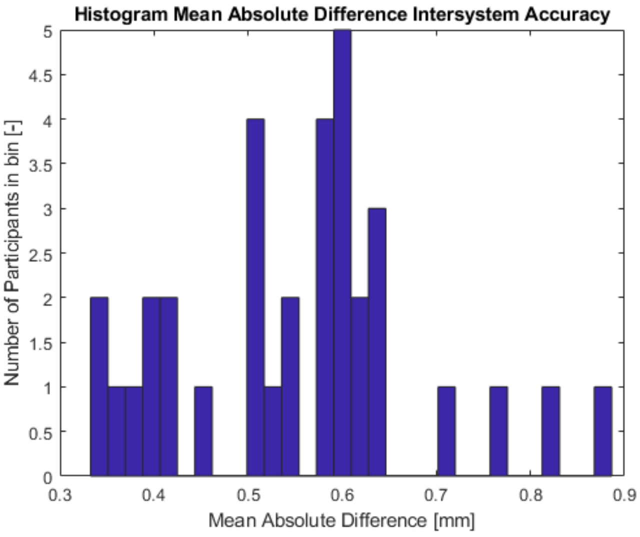

(a)

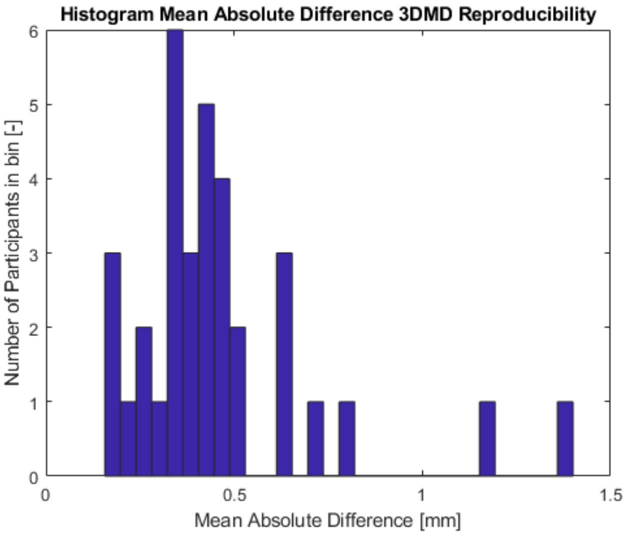

(b)

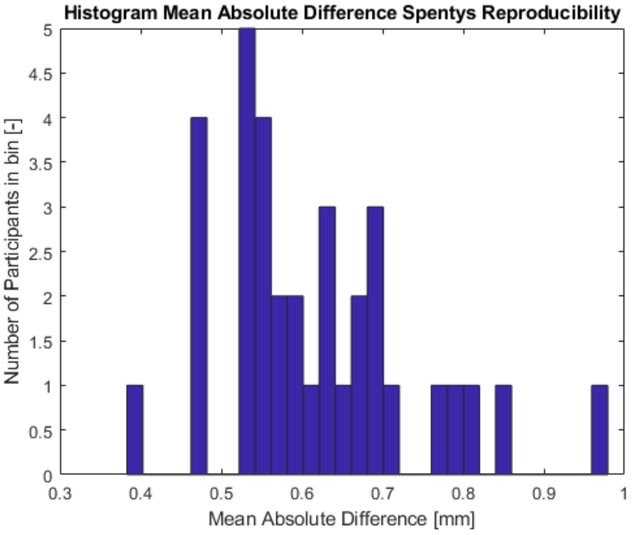

(c)

Figure 7: Histograms of the Mean Absolute Difference of: (a) the Intersystem Accuracy, (b) the 3dMD Repeatability and (c) the Spentys Repeatability
